# Healthcare worker acute respiratory illness cluster in 2020: Could it be from COVID-19?

**DOI:** 10.1017/ice.2020.364

**Published:** 2020-07-23

**Authors:** Win Mar Kyaw, Aung Aung Hein, Zoe Zhang Xiaozhu, Lay Tin Lee, Cui Lin, Brenda Ang, Angela Chow

**Affiliations:** 1 Department of Clinical Epidemiology, Tan Tock Seng Hospital, Singapore; 2 Occupational Health Department, Tan Tock Seng Hospital, Singapore; 3 National Public Health Laboratory, Ministry of Health, Singapore; 4 Infection Prevention and Control Unit, Tan Tock Seng Hospital, Singapore; 5 Lee Kong Chian School of Medicine, Singapore

*To the Editor—*Since the emergence of coronavirus disease 2019 (COVID-19) caused by severe acute respiratory coronavirus virus 2 (SARS-CoV-2) in China, >45,000 confirmed cases including >60 healthcare workers (HCWs) have been reported in Singapore.^1,2^ Healthcare workers (HCWs) are at increased risk of nosocomial COVID-19 infection.^3^ In 2003, almost one-third of ward-based HCWs at Tan Tock Seng Hospital (TTSH) in Singapore were infected with the severe acute respiratory syndrome (SARS) from an index patient.^4^ After the SARS nosocomial outbreak, web-based staff sickness surveillance systems have been established at TTSH for the early detection of HCW clusters of acute respiratory infection (ARI).^5–7^ Additionally, a risk-based approach to the use of personal protective equipment (PPE) by HCWs, with full PPE donned in high-risk areas and minimally surgical masks in low-risk areas were implemented.^7^

During the COVID-19 pandemic, a team of public health-trained personnel maintained close monitoring of staff sickness reporting to identify ARI clusters among the 12,000 HCWs working at the 1,600-bed TTSH and its collocated 330-bed National Centre for Infectious Diseases, the national referral centre for COVID-19 response. We examined the epidemiology of ARI clusters identified in HCWs in the first 27 weeks of 2020, and we compared them with the ARI clusters in 2019. An ARI cluster was defined as ≥3 HCWs or ≥5% of the total staff strength (whichever was higher) from the same work location reporting ARIs within 4 consecutive days. Each ARI cluster was followed-up with active case findings and infection prevention measures, including enhanced hand hygiene and enforcement of adherence to appropriate PPE and referral of sick staff to the in-house occupational health clinic (OHC). Nasal and throat swabs were taken from symptomatic HCWs who worked at the OHC. Samples were sent to the National Public Health Laboratory for influenza polymerase chain reaction (PCR) and respiratory multiplex PCR (FilmArray Respiratory Panel 2) tests. SARS-CoV-2 PCR testing was implemented in 2020 at the start of the COVID-19 pandemic.

**Fig. 1.**
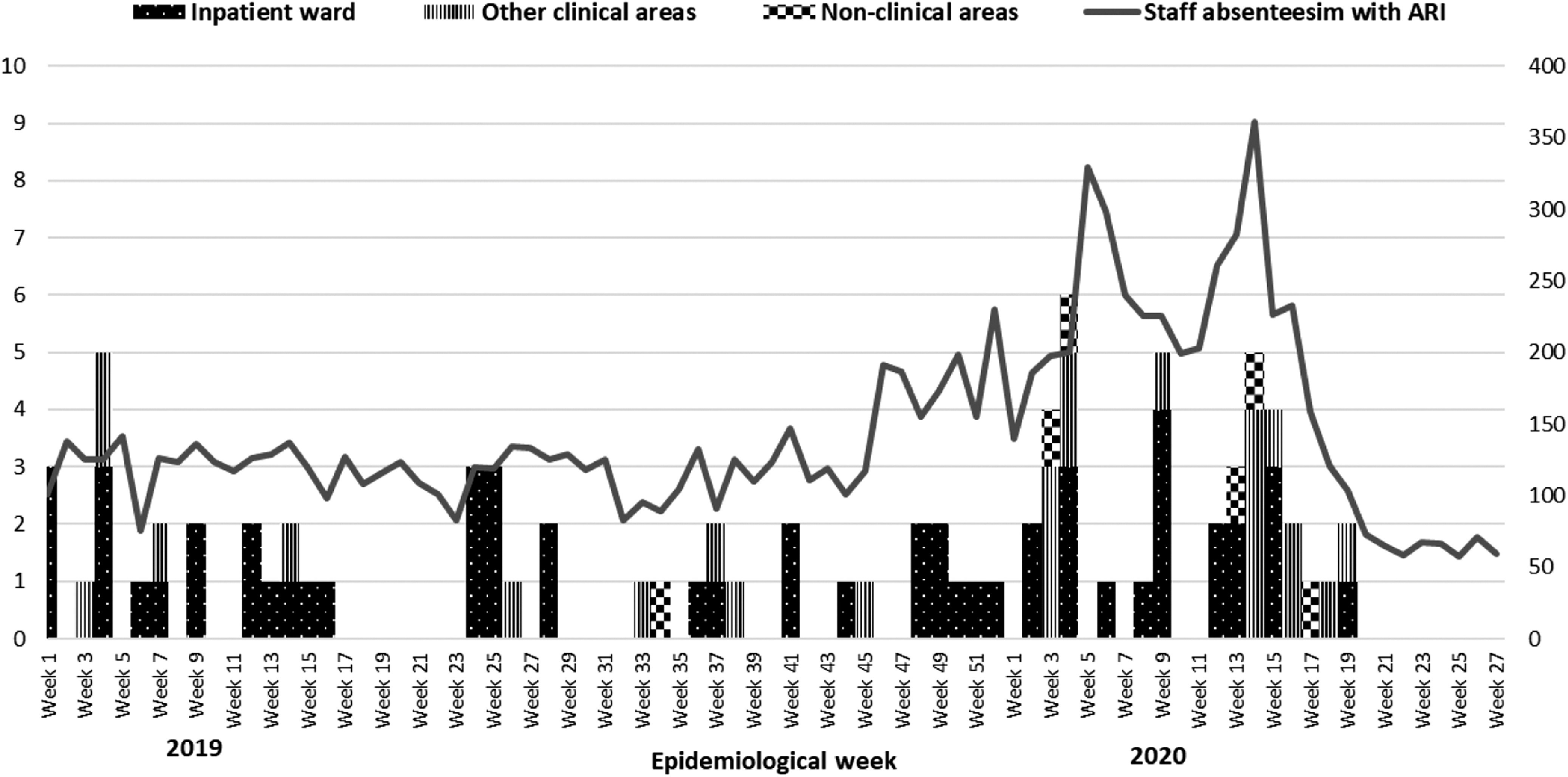
Staff acute respiratory infection (ARI) clusters (primary axis) and staff absenteeism from ARI (secondary axis) Tan Tock Seng Hospital (TTSH) and National Centre for Infectious Diseases (NCID) (epidemiological week 1 in 2019 to epidemiological week 27 in 2020).

During the 79-week study period, the mean weekly number of staff absences due to ARI was 142 (standard deviation [SD], 62). More than half of the ARI clusters were identified among HCWs who worked in inpatient wards (n = 55, 64%). Other clinical areas (eg, pharmacy, physiotherapy, operating theatres, and outpatient clinics) accounted for more than one-quarter of the clusters (n = 25, 29%). The remaining clusters were identified among HCWs who worked in nonclinical areas (n = 6, 7%) (Fig. 1). In total, 47 ARI clusters were identified in the entire year of 2019, and 39 clusters were identified in first 27 weeks of 2020.

Compared to the first 27 weeks of 2019 (n = 28), the number of ARI clusters identified among staff working in inpatient wards in 2020 (n = 39) was significantly lower: 49% versus 78%, respectively (OR, 0.26; 95% CI, 0.09–0.78; *P* = .016). Median cluster sizes were slightly larger: 8 (IQR, 6–21) for 2020 versus 7 (IQR, 4–10) for 2019 (OR, 1.07; 95% CI, 0.99–1.15; *P* = .067). Median cluster duration was longer in 2020 than in 2019: 22 days (IQR, 13–40) versus 19 days (IQR, 13–25), respectively (OR, 1.02; 95% CI, 0.98–1.06; *P* = .290). Among ARI clusters, almost twice the number of clusters in 2020 had at least 1 respiratory pathogen identified (n = 11, 28%) compared with 2019: 11 (28%) versus 6 (21%), respectively (*P* = .531).

Rhinovirus was the most common viral pathogen detected in both years: 6 clusters (15%) in 2020 and 3 clusters (11%) in 2019. This possibly reflects the most common circulating noninfluenza viral pathogen among ARI episodes in the community.^8^ Human coronaviruses 229E/HKU1/OC43 were detected in both years: 4 clusters (10%) in 2020 and 1 cluster (4%) in 2019. Adenovirus was identified in 8% of ARI clusters in 2020, although it was not detected in any of the ARI clusters in 2019. Parainfluenza viruses (5%) were also detected during the first 27 weeks of 2020 but not in 2019. Influenza viruses were detected in 2 clusters in 2019 but in none of the clusters in 2020. SARS-CoV-2 virus was not detected in any of the HCW ARI clusters in 2020.

Since start of the pandemic, despite an increase in ARI clusters detected, SARS-CoV-2 has not been detected. This absence reflects the adequate protection of HCWs from acquiring SARS-CoV-2 infection in the hospital. Notably, no pathogen was identified in HCW ARI clusters after epidemiological week 14 in 2020, and a downward trend of the weekly number of staff ARIs reported from epidemiological week 17. No staff ARI cluster was identified after epidemiological week 19. These trends are likely the consequence of hospital-wide enhanced infection prevention measures (eg, safe distancing, having meals alone, and the donning of surgical masks at all times in all hospital areas) instituted since epidemiological week 15 in 2020 (ie, the week ending April 11, 2020).

Close surveillance of staff absenteeism due to ARI and epidemiological investigations of HCW ARI clusters with screening for respiratory viruses and SARS-CoV-2 are crucial as COVID-19 pandemic emergency responses relax, economic activities resume, and travel bans are lifted. Because it is unlikely that COVID-19 infections will taper off soon around the world, countries should consider having all HCWs wear surgical masks at all times in healthcare settings.
